# Modeling the Fluid Dynamics in a Human Stomach to Gain Insight of Food Digestion

**DOI:** 10.1111/j.1750-3841.2010.01748.x

**Published:** 2010-09

**Authors:** MJ Ferrua, RP Singh

**Affiliations:** Authors are with Riddet Inst., Massey Univ.Palmerston North, New ZealandAuthor Singh is also with Dept. of Biological and Agricultural Engineering. Univ. of CaliforniaDavis, CA 95616, U.S.A.

**Keywords:** computational fluid dynamics (CFD), gastric flow, gastric motility, stomach model, viscosity

## Abstract

During gastric digestion, food is disintegrated by a complex interaction of chemical and mechanical effects. Although the mechanisms of chemical digestion are usually characterized by using *in vitro* analysis, the difficulty in reproducing the stomach geometry and motility has prevented a good understanding of the local fluid dynamics of gastric contents. The goal of this study was to use computational fluid dynamics (CFD) to develop a 3-D model of the shape and motility pattern of the stomach wall during digestion, and use it to characterize the fluid dynamics of gastric contents of different viscosities. A geometrical model of an averaged-sized human stomach was created, and its motility was characterized by a series of antral-contraction waves of up to 80% relative occlusion. The flow field within the model (predicted using the software Fluent™) strongly depended on the viscosity of gastric contents. By increasing the viscosity, the formation of the 2 flow patterns commonly regarded as the main mechanisms driving digestion (i.e., the retropulsive jet-like motion and eddy structures) was significantly diminished, while a significant increase of the pressure field was predicted. These results were in good agreement with experimental data previously reported in the literature, and suggest that, contrary to the traditional idea of a rapid and complete homogenization of the meal, gastric contents associated with high viscous meals are poorly mixed. This study illustrates the capability of CFD to provide a unique insight into the fluid dynamics of the gastric contents, and points out its potential to develop a fundamental understanding and modeling of the mechanisms involved in the digestion process.

**Practical Application:**

This study illustrates the capability of computational fluid dynamic techniques to provide a unique insight into the dynamics of the gastric contents, pointing out its potential to develop a fundamental understanding and modeling of the human digestion process.

## Introduction

New trends in consumer preferences and attitudes toward foods have significantly impacted the food market, creating unique opportunities for a new generation of products aimed to provide health benefits beyond basic nutrition (Gray and others 2003). During the last decade, the functional food market has been identified as one of the fastest-growing markets in the sector, and it is expected to shape the future of the global food industry (Norton and others 2006). However, to ensure its successful development and long-term survival, it is essential to enhance the consumer confidence and acceptance of functional foods by developing more specific and scientifically supported health claims (Childs and Poryzees 1998; Gray and others 2003).

To manufacture future foods, with scientifically supported health claims, it is essential to develop an underpinning knowledge and understanding of how food components and structures are transformed and absorbed during digestion (Norton and others 2006).

The human digestive system (GI tract) consists of a series of specialized organs and glands, each of them playing a specific role in the digestion and/or absorption of the meal. During digestion, the food structure is broken down by a complex interaction of chemical and mechanical processes, triggered by the secretionary and motor response of the GI tract. Digestive juices are secreted to promote the enzymatic splitting of proteins, carbohydrates, and fats, while muscle contractions of the entire GI tract generate the mechanical forces and fluid motions that promote not only the mechanical breakdown of the food, but also its chemical digestion, absorption, and transport. These secretionary and motor responses of the GI tract are significantly affected by the individual, digestion time, and the amount, composition, and physicochemistry of the meal (Coupe and others 1991; Camilleri and Prather 1993; Mayer 1994; Parada and Aguilera 2007). This variability, together with the complex interaction and difficult characterization of the chemical and mechanical processes involved, has prevented a good understanding of the process.

Since the beginning of the 1990s, a series of *in vitro* systems have been developed to analyze human digestion (Aoki and others 1992, 1993; Molly and others 1993; Minekus and others 1995; Oomen and others 2003; Mainville and others 2005; Kong and Singh 2008b; Wickham and others 2009). However, despite the use of pharmacological, physiological, and biochemical knowledge of the human and animal GI tract, conflicting results have been obtained (Yoo and Chen 2006). One of the main drawbacks of this approach is the difficulty of reproducing the geometry and motility of the GI tract. So far, the possibility of developing an *in vitro* system capable of accurately reproducing the fluid-mechanical forces that promote digestion is still extremely difficult to achieve, if not impossible.

Advanced fluid dynamics programs offer a promising technique to characterize the mechanisms promoting digestion (Singh 2007). Based on the motor response of the GI tract and the physicochemical properties of luminal contents, computational fluid dynamics (CFD) can be used to numerically model the dynamics of gastrointestinal contents during digestion. Although some initial attempts were done to simulate the gastric flow during digestion (Pal and others 2004; Pal and others 2007), the computational effort required to reproduce the geometry and motility of the stomach prevented a good characterization of the system. So far, only a 2-D analysis has been informed in the literature.

The goal of this study was to use CFD techniques to develop a 3-D model of the geometry and motility of the human stomach during digestion, and use it to characterize and compare the fluid dynamics of gastric contents of different viscosities.

## Materials and Methods

### Stomach geometry and motility during digestion

#### Stomach geometry and size

**Figure 1:**
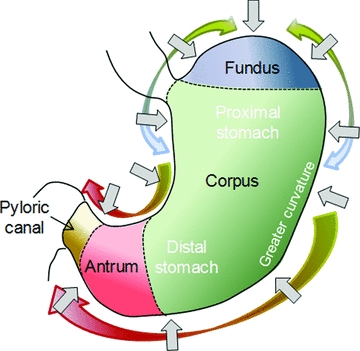
A schematic diagram of a human stomach.

The human stomach is a “J”-shaped, hollow and elastic organ, capable of dilating to accommodate more than 1-L meal without a significant increase in its luminal pressure (Figure 1). Although recent advances in medical imaging technologies has allowed a more accurate description of the stomach geometry at various physiological states (Liao and others 2004; Pal and others 2007), no unique description of the size or shape of the stomach can be specified.

The normal capacity of the human stomach varies from 0.25 to about 1.7 L (Einhorn 2009), and its geometry not only changes from one individual to another, but it is also significantly influenced by the position of the body, the condition of surrounding viscera and organs, the amount and type of meal ingested, and the digestion time (Liao and others 2004; Schulze 2006). After a typical meal, an average-sized human stomach is about 10 cm wide at its widest point, its greater curvature is about 30 cm long, has a pyloric ring diameter of 1.1 cm or less, and its average capacity is about 0.94 L (Keet 1993; Schulze 2006).

#### Gastric motility during digestion (“postprandial” period)

The motor activity that develops in response to the ingestion of a meal has a critical role in gastric digestion. It not only develops the fluid-mechanical forces promoting the mechanical and chemical digestion of the food, but it also allows the stomach to act as a holding chamber by receiving and storing the ingested meal. These particular responses of the stomach walls are commonly known as “adaptive relaxation” and “receptive relaxation,” respectively (Schwizer and others 2002). In addition, it is also suspected to control the release of gastric contents into the duodenum, once their physicochemical properties render suitable for the next phase of digestion (Pal and others 2004). In general, the motility pattern of the gastric wall can be characterized by 2 types of muscle contractions, as discussed in the following.

The 1st type of motor activity originates and develops in the upper part of the stomach (Figure 1). It is characterized by slow and weak wave activities that only produce a slight indentation of the stomach wall (Pal and others 2007; Lammers and others 2009).

The 2nd type of motor activity is characterized by a series of regular-peristaltic antral contraction waves (ACW) that originate at the middle of the stomach and propagate circumferentially toward the pylorus (Figure 1). These contractions are born as shallow indentations, but they deepen as they travel down (virtually obstructing the stomach lumen as they approach the pylorus). Their frequency approximates 3 cycles per minute and they are controlled by the electrical stimulation generated by a gastric pacemaker localized to an area in the midcorpus along the greater curve of the stomach (Marciani and others 2001b; Pal and others 2004; Schulze 2006; Stern and Koch 2008). Their propagation velocity increases from the proximal to the distal stomach, and they are expected to move slower along the inner curvature. They are also presumed to indent the inner curvature less than the greater one (Camilleri and Prather 1993; Mayer 1994).

The 3rd type of motor activity can be described as a tonic contraction of the entire gastric wall, which allows the stomach to accommodate itself to varying volumes (Figure 1).

The specific motility pattern of these contractions during the digestion process is not unique. The motor response of the stomach is carefully regulated by a series of complex and interdependent neuro-hormonal mechanisms, which in turn are controlled by the amount, composition, and physicochemical properties of the meal (Keinke and others 1984; Coupe and others 1991; Camilleri and Prather 1993; Mayer 1994; Horowitz and others 1994; Indireshkumar and others 2000; Camilleri 2006).

Due to experimental limitations, the motility pattern of the stomach wall is still not fully characterized. Novel imaging techniques (such as real time ultrasonography and echoplanar MRI) have been identified as promising techniques to monitor gastric motility during digestion (Marciani and others 2001b; Hausken and others 2002; Pal and others 2004; Schulze 2006). But so far, these techniques have been only used to analyze the motility pattern of the ACW activity, for a limited number of Newtonian liquid meals.

A detailed characterization of the ACWs was performed by Pal and others (2004, 2007). By using MRI analysis, they analyzed the ACW activity that develops for 20 min after the ingestion of 500 mL of a glucose solution (10%, w/w). They found that, on average, the ACWs were initiated every 20 s at about 14.4 cm from the pylorus. The average width of the ACWs was determined to be 1.8 cm, and their velocity was informed in terms of a linear propagation value of 0.25 cm/s. The relative occlusion of the ACW increased as the wave travels from the proximal to the distal stomach. During the first 17.5 s of the ACW life, its relative occlusion linearly increased from 0% to 40%. After that, it remained constant for 16 s, to start linearly increasing again during the last 24 s of its life (reaching a value of up to 90% at the pylorus).

### Computational model used to model gastric digestion

#### Computational model of an average-sized human stomach

The significant variability in the shape and size of the stomach, together with the lack of a good experimental characterization of its motor activity, does not justify the development of an algorithm capable of deforming the intricate geometry of an accurate model of a real stomach. Instead, we created a simplified 3-D model, capable of reproducing the shape and dimensions of an averaged human stomach, as follows.

**Figure 2:**
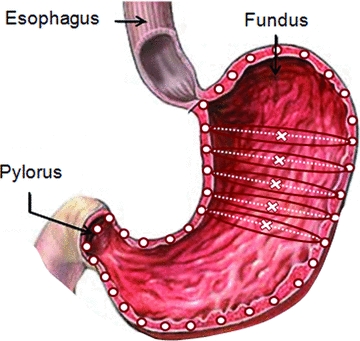
Two-dimensional image used to develop the geometrical model of an average-sized human stomach.

**Figure 3:**
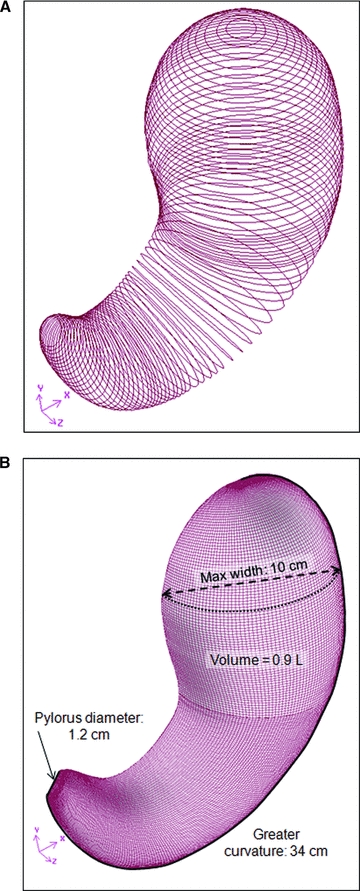
Construction of a 3-D model of the average human stomach. (A) Series of circles used to develop the 3D geometry of the stomach model. (B) Isometric view of the final geometrical model.

The shape of the plane that bisects the stomach along its lesser and greater curvatures was outlined by drawing a series of points on the perimeter of a typical image of the human stomach (MedlinePlus Health) (Figure 2). The coordinates of these points were imported into a 3-D drawing software, Gambit 2.4.6 (Anonymous 2007). A series of segments were then created by uniting corresponding pairs of points located along the lesser and greater curvature of this 2-D contour. By determining the middle point of each of these segments, a series of circles were created (Figure 2). The 3-D model of the stomach was then developed by using these circles as a base frame (Figure 3A).

By considering the symmetry of the stomach model with respect to the bisectional plane, the final computational domain consisted in only one half of the entire stomach model (Figure 3B). The computational mesh was created using a structured Cooper scheme. It was composed primarily of hexahedral elements, with a size of 0.1 cm in the lower region of the stomach and 0.14 cm in the upper one. The final mesh consisted of 767,800 elements, with 70% of them in the antropyloric region of the stomach (Anonymous 2007).

The capability of the model to represent the geometry of an averaged-sized human stomach was confirmed by comparing its characteristic dimensions against those obtained from the literature (Table 1).

**Table 1:** Characteristic dimension of the 3D geometrical model developed compared with the average-sized human stomach.

Characteristic length	Developed model	Average-sized stomach^a^
Greater curvature length (cm)	34	30
Widest section wide (cm)	10	10
Pyloric ring diameter (cm)	1.2	1.1
Volume capacity (L)	0.9	0.94

a From Keet (1993) and Schulze (2006).

#### Numerical modeling of gastric motility during digestion

The motility pattern of the stomach was simulated using a numerical algorithm that identified and relocated each node of the computational domain as a function of time.

Due to the lack of information, the gastric motility modeled in this study was based on the ACW activity experimentally analyzed by Pal and others (2004, 2007). However, it is noteworthy that to fully characterize the propagation of the ACWs, some additional parameters needed to be defined. These parameters comprised not only the directions in which the ACWs deform the gastric wall, but also the directions in which the average velocity and width of the ACW (informed in the literature) were determined.

**Figure 4:**
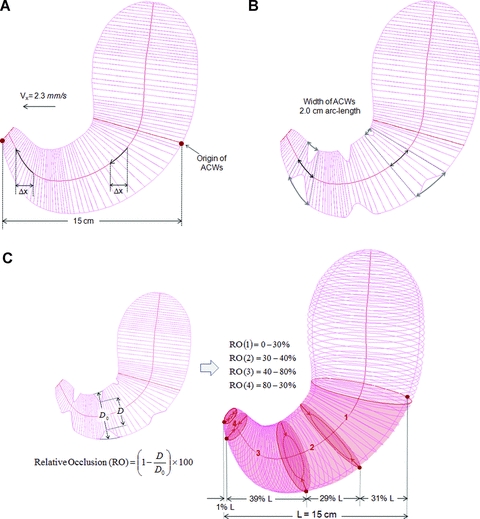
Motility pattern of the ACWs during digestion. (A) Origin and average-velocity of ACWs. (B) Width of the ACWs. (C) Direction and amplitude of the ACWs.

Based on the general characteristics of the ACW activity (section “Gastric motility during digestion (“postprandial” period)”), the motility pattern of the stomach wall was characterized as follows. The ACWs were initiated every 20 s at 15 cm from the pylorus with a life span of 58 s (Figure 4A). They were assumed to propagate at a constant velocity of 2.3 mm/s along the horizontal axis within the symmetry plane of the stomach model (Figure 4A). This assumption, together with the J-shape of the stomach, ensured the acceleration of the ACWs in the antropyloric region and their faster propagation along the greater curvature with respect to the lesser one. In particular, the average velocity of the ACW (± its standard deviation) was 4.6 ± 1.5 mm/s (varying from 2.7 to 8.0 mm/s), while along the lesser curvature it was 2.1 ± 1.0 mm/s (varying from 1.0 to 5.2 mm/s). The width of the ACWs was assumed to be 2.0 cm along the center line of the stomach model; assumption that ensured that the ACWs indent the greater curvature more than the lesser one (Figure 4B). Finally, the ACWs were assumed to deform the gastric wall circumferentially, following the plane of the auxiliary circles used to develop the 3-D model of the stomach (Figure 4C). Their relative occlusion increased from 0% to 80%, as illustrated in Figure 4C.

As expected, the propagation of the ACWs causes a variation on the capacity of the stomach domain. Since the stomach was modeled as a closed system with incompressible gastric contents, to maintain the capacity of the stomach constant (that is, ensure continuity), a series of tonic contractions of the upper wall of the stomach were defined. Due to the lack of experimental information, the motility pattern of these contractions was, to some extent, arbitrarily defined. Similar to the ACWs, these tonic contractions deformed the gastric wall circumferentially, following the direction of the auxiliary circles created to develop the 3-D model of the stomach. The percentages of contraction/expansion of the upper wall, at each instant of time, linearly increased from zero (at the mid corpus) to a maximum value (at the top of the fundus region). These maximum values were specifically computed to ensure that the capacity of the stomach model remained constant and reached values not higher than 8%.

**Figure 5:**
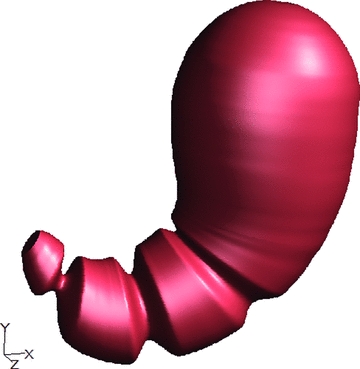
Image of the motility pattern of the gastric wall as numerically simulated.

The type of deformation imposed by the motility pattern modeled in this study is illustrated in Figure 5.

### Flow model

#### Governing equations

The flow field that develops within the stomach was modeled as a laminar and incompressible fluid flow of a continuous liquid phase (Pal and others 2004, 2007). Under these flow conditions, the conservation of mass and momentum within the system were given by Eq. 1 and 2, respectively.
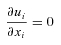
(1)
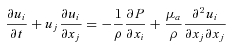
(2)

#### Physicochemical properties of gastric digesta

Since the motor activity modeled in this study is associated with the ingestion of a 10% glucose solution meal, the gastric content was assumed to be a Newtonian fluid with a density of 1 kg/L and a viscosity of 10^−3^ Pa.s (Schmidt and others 1981; Buia and Nguyen 2004).

However, it is noteworthy that although liquid foods exhibit a wide range of rheological properties, only a few of them exhibit a Newtonian behavior. Among these foods, even fewer have a viscosity of the same order of magnitude than water, with viscosities reaching values of up to 10 Pa.s (Steffe 1996). In general, the addition of even a small amount of a dissolved polymer (approximately 1%) can substantially increase the viscosity of the solution (Rao 2007).

Due to the laminar behavior of the fluid flow and the proximity of the gastric walls, the flow field that develops within the stomach can be significantly affected by the rheological properties of the gastric content, and in particular by its viscosity. To investigate this effect, the flow field developed within the stomach model in the case of a Newtonian fluid with a viscosity 1000 times higher (that is, 1 Pa.s) was analyzed. An example of a Newtonian liquid food with a viscosity of this order of magnitude is honey (Steffe 1996).

#### Boundary conditions

**Figure 6:**
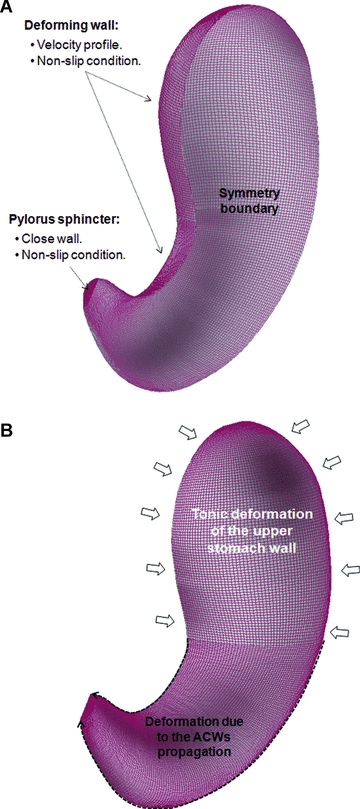
Boundary conditions. (A) Boundary types. (B) Deformation of stomach walls.

Due to the elliptic nature of the Navier–Stokes equations in the space domain, boundary conditions had to be prescribed around the entire geometrical model (Figure 6).

To correctly simulate the flow behavior at the moving boundaries, the velocity profile of the deforming wall needed to be numerically modeled at each instant of time, and prescribed together with the nonslip condition of the fluid flow.

Under the scope of this study, neither the roughness of the stomach wall nor the presence of the mucous layer on top of it was considered. Although the roughness of the wall may potentially affect the flow behavior within the system, its effect on the laminar flow within a macroscale system has been traditionally assumed to be negligible in the case of Newtonian fluids (Engin and others 2004). Based on this observation, the small scale of the roughness length of stomach lining (McMahon and others 2007), and the presence of the mucous layer on top of it, the effect of the roughness of the wall on the fluid dynamics of gastric contents was assumed negligible. However, it is worthwhile to mention that the presence of the mucous layer may affect the nonslip condition at the stomach wall. As far as we know, there is no scientifically based information addressing this particular point, and future work needs to be done to characterize the boundary condition at the stomach wall.

Gastric contents empty into the duodenum whenever a positive gastroduodenal pressure gradient is established in the presence of an open pylorus (Hausken and others 2002; Schulze 2006). The rate and pattern of gastric emptying is regulated by the motor response of the GI tract, which in turn is regulated by a series of complex and interdependent neurohormonal mechanisms that are triggered by the physicochemical properties of luminal contents and a series of neurophysiological reflexes involving duonenal absorption (Keinke and others 1984; Horowitz and others 1994; Indireshkumar and others 2000; Camilleri 2006). So far, the difficulty in measuring luminal pressures and transpyloric flows, in relationship with the motility pattern of the gastrointestinal walls, has limited the knowledge required to fully understand and characterize the kinetics of gastric emptying. Although different mathematical models have been proposed in the literature (Versantvoort and others 2004; Hellström and others 2006; Kong and Singh 2008a), none of them actually considers the effect of the physicochemical properties of the meal, the pulsative pattern of gastric emptying or the reflux episodes experimentally determined by several *in vivo* studies (King and others 1984, 1987; Hausken and others 1992; Hausken and others 2002).

Based on these observations, the emptying of gastric contents was not modeled at this stage of the work. The numerical model was only used to get a better understanding of the flow field that develops within the stomach at the beginning of the digestion process.

### Numerical solution of the flow model

The CFD software Fluent™ 6.3.26 (ANSYS, Inc., Canonsburg Pa., U.S.A.) was used in conjunction with a desktop computer (CPU Intel Core i7 940 2.93 GHz, 3.23 GB of RAM) to numerically deform the computational domain of the stomach and solve the transient flow field that develops within it.

The flow model within the system was solved using an implicit pressure-based coupled algorithm (Anonymous 2007). By using the pressure-based solver the continuity of the velocity field is enforced by means of a pressure equation that is derived from the continuity and the momentum equations defined in section “Governing equations.” The velocity and pressure fields were then computed by solving the momentum and pressure equations in a coupled fashion. The PRESTO! interpolation scheme was used to compute the face values of pressure from the cell values (Anonymous 2007). The discretization of the convective and diffusive terms of the momentum equation was achieved by using a 2nd-order upwind algorithm and the Green–Gauss cell-based algorithm, respectively. The governing equations were discretized in time by using a 1st-order implicit formulation. The criteria used to judge the convergence of the numerical solution required a decrease in the value of the scaled residual to 10^−4^ for the velocities and to 10^−3^ for continuity (Anonymous 2007). An adaptive time stepping method was used to solve the transient behavior of the flow field. The time step varied along the simulation depending on the relative occlusion caused by the ACW and the viscosity of the fluid. In general, it varied between 0.1 and 0.01 s for the low viscosity fluid, and between 0.05 and 0.005 s in the case of the high viscous one.

Since the temperature of the meal affects the emptying rate from the stomach (Bateman 1982; Troncon and Iazigi 1988; Mishima and others 2009), it is expected that the corresponding temperature of gastric contents varies during the digestion until reaching the human's core temperature. However, due to the lack of substantial information to perform a numerical analysis of the energy transfer process within the human stomach, the fluid dynamics of gastric contents were modeled under isothermal conditions.

At time zero, no motor activity was prescribed in the walls of the stomach model and the fluid within it was assumed at rest. The deformation of the stomach wall was then initiated, and the flow field within the system was simulated until a periodic solution was reached. Based on the gastric motility modeled, the motility pattern of the stomach wall becomes periodic after 38 s of simulated real time, repeating itself every 20 s.

## Results and Discussion

### Flow field behavior within the stomach

**Figure 7:**
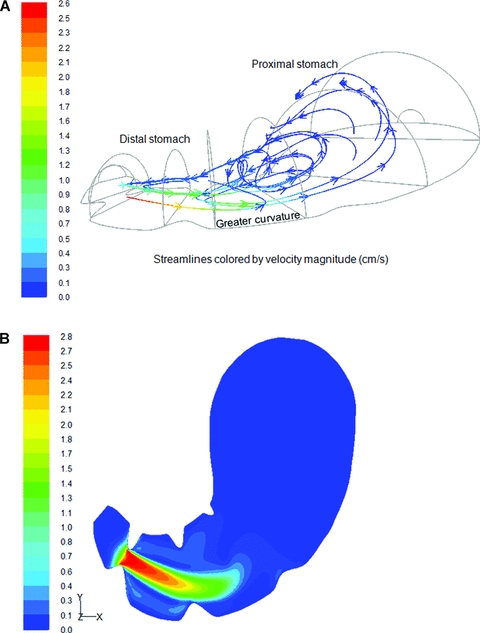
Gastric flow at *t*+ 10 s for a Newtonian fluid (0.001 Pa.s). (A) Streamlines of the fluid flow, colored by velocity magnitude (cm/s). (B) Contour of the velocity field in the middle plane of the stomach model.

**Figure 8:**
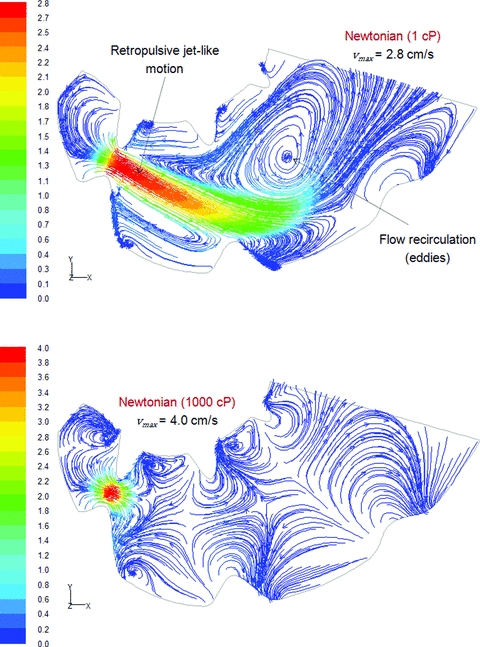
Effect of viscosity on the formation of the retropulsive-jet like motion and eddy structures. Streamlines of the fluid flow within the stomach's middle plane at *t*+ 10 s, colored by velocity magnitude (cm/s).

As a result of the motor activity of the gastric wall, a highly 3-D flow was predicted within the stomach, independently of the viscosity of the fluid. The strongest fluid motions were also always predicted in the lower part of the stomach, while a slow recirculation of gastric contents from the proximal to the distal stomach was also identified. These results (illustrated in Figure 7 in the case of the 0.001 Pa.s fluid) are in good agreement with the classical description of gastric functions, where the upper part of the stomach acts as a reservoir and supplier of gastric contents to the antrum, whereas the lower part is responsible for the mechanical forces and fluid motions that promotes their breakdown and mixing (Pal and others 2007; Kong and Singh 2008a). However, unlike these overall flow patterns, the local characteristics of the flow field within the stomach were significantly influenced by the viscosity of the fluid (Figure 8).

**Figure 9:**
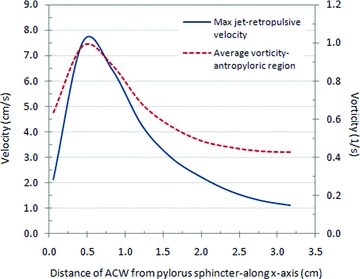
Quantitative characterization of the retropulsive jet and eddy structures that developed within the antropyloric region, as the ACWs propagate toward the pylorus (Newtonian fluid, 0.001 Pa.s).

The flow field predicted by the model, in the case of the ingestion of a 10% glucose solution (Newtonian, 0.001 Pa.s), was in good agreement with the classical description of gastric motions (Schulze 2006). In particular, the flow field predicted within the antropyloric region was characterized by 2 main flow patterns (Figure 8). As the ACWs moved toward the pylorus, gastric contents were forced back, and a retropulsive jet-like motion developed once the gastric occlusion reached a value of 56% (Boulby and others 1999). To illustrate and quantify the development of this jet-like motion, the maximum retropulsive velocity was tracked during the 20 s period of the flow (Figure 9). As the ACW approaches the pylorus sphincter, the increasing speed and occlusion of the ACW strengthened this retropulsive jet successively faster, reaching a maximum retropulsive velocity of 7.6 cm/s in the most occluded section of the pylorus canal. The other main flow pattern identified within the antrum region corresponded to the formation of circular motions (eddies) between 2 consecutive ACWs (Figure 8). Alike the retropulsive jet, the strength of these eddies, characterized by the average-vorticity of the flow field, increased as the ACWs approaches the distal antrum (Figure 9).

By forcefully mixing, rubbing and grinding of gastric contents, the retropulsive jet-like motion and eddy structures have been largely regarded as the main causes driving the gastric digestion of the food (Pallotta and others 1998; Pal and others 2004; Schulze 2006). However, the numerical results suggested that the characteristics, as well as the development, of these particular flow features are significantly affected by the rheological properties of the fluid.

By increasing the viscosity of gastric contents to 1 Pa.s, higher retropulsive velocities were predicted within the system, but the development of a retropulsive jet-like motion is arguable (Figure 8). Similar to the case of a low viscous fluid, the higher retropulsive velocities were predicted at the location of the ACW peak, but they were confined to a smaller region at the core of the contracted section of the stomach. This result may be explained by the increased action of viscous stresses, which together with the stationary conditions of the gastric wall at the ACW peak, slow down the flow even further away from the wall. Since the deformation of the gastric wall and fluid density were assumed equal to those of the low viscous case, larger retropulsive velocities should develop at the core of the luminal region to ensure continuity. Similarly, the almost immediate decay of the retropulsive motion away from the ACW may be explained by the enhanced diffusion of viscous effects together with the presence of stationary walls between consecutive ACWs.

To quantify the effect of the rheological properties of the fluid on the characteristics of the jet, the initial velocity and length of the strongest retropulsive jet-structure developed within the system were computed (Table 2). The length of the jet was defined along its axis as the distance from its origin to the point where the jet-velocity decays 40% (largest decay that allowed the characterization of the jet-like structure's length before it reached the largest curvature of the stomach wall).

**Table 2:** Effect of the rheological properties of the fluid on the jet-like structure.

Jet's characteristics	Newtonian (1 × 10^−3^Pa.s)	Newtonian (1 Pa.s)
Initial velocity (cm/s)	7.5	11.9
Length (cm)	2.3	0.2

**Figure 10:**
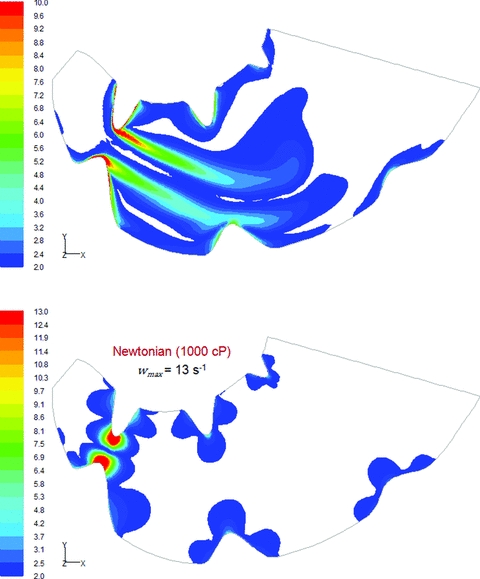
Effect of viscosity on the vorticity of the flow field. Contour of vorticity within the stomach middle plane at *t*+ 10 s (lower part of the stomach).

**Figure 11:**
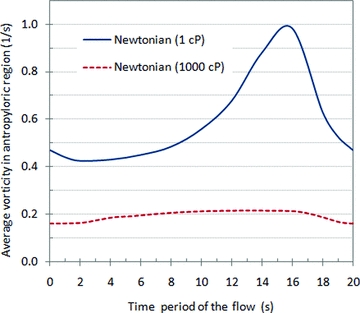
Effect of the rheological properties of gastric contents on the average value of the vorticity field predicted within the antropyloric region.

As illustrated in Figure 8, the formation and strength of eddy structures were also affected by viscosity. By increasing the viscosity of the fluid, the development of circular motions was confined to regions closer to the ACWs, and a lower and more uniform vorticity field was predicted (Figure 10). To quantify the effect of rheological properties on the strength of the eddy structures, the average value of the vorticity field predicted within the antropyloric region was tracked during the 20 s period of the flow (Figure 11).

These results suggest that, contrary to the traditional idea of a complete and rapid homogenization, gastric contents associated with high viscous meals seem to be poorly mixed. This result was also experimentally observed by Marciani and others (2001a), where the process of dilution and mixing of gastric contents associated with viscous meals were analyzed by using MRI color-coded dilution maps.

To analyze the effect of modeling the 3-D feature of the system, the flow field predicted by Pal and others (2004) for a 1 Pa.s Newtonian fluid was compared against the one predicted in this study. The results indicated that although the velocity or vorticity profiles obtained within the symmetry plane of the stomach are not significantly affected, their magnitudes are. In particular, the use of an actual model of the system predicted velocity and vorticity values larger by one order of magnitude.

#### Experimental insight into the performance of the flow model

The experimental characterization of the flow field within a complex, deforming wall-bounded system (as the one modeled in this study) is extremely difficult to achieve. To obtain an insight into the performance of the flow model, a nonintrusive flow measurement technique called particle image velocimetry (PIV) was used to characterize the flow field that develops within a closed chamber due to the peristaltic deformation of one of its walls.

**Figure 12:**
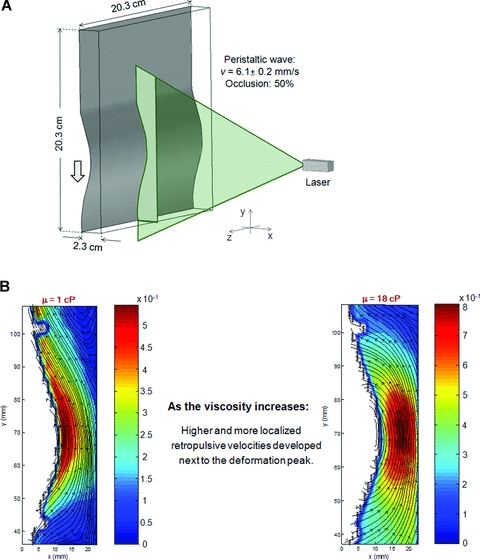
(A) Experimental system used to measure the flow field that develops within an acrylic chamber due to the peristaltic deformation of one of its walls. (B) Instantaneous streamlines of the velocity fields experimentally measured using PIV (cm/s).

The experimental system consisted of an acrylic chamber (20.3 × 20.3 × 2.3 cm), where one of the walls was substituted by a neoprene sheet (Ferrua and others 2009). The flow within the chamber was developed by periodically moving a cycloid-shaped object along the neoprene sheet, thus creating a longitudinal hump (Figure 12A). To approximate the ACW activity modeled in this study, the hump was designed to impose a 50% occlusion across the chamber height and it was moved at a speed of 5.5 ± 0.5 mm/s. The flow field that developed within the chamber was measured for 2 different Newtonian fluids: water (0.001 Pa.s) and Johnson & Johnson baby oil (0.020 Pa.s).

As illustrated in Figure 12B, the experimental data confirmed that the higher retropulsive velocities developed at the location of the contraction and (similar to the results predicted within the stomach model) as the viscosity of the fluid increases, higher and more localized retropulsive velocities developed at the core of the luminal section contracted by the wave.

### Pressure fields within the stomach

**Figure 13:**
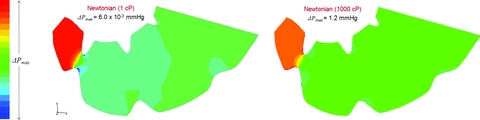
Effect of viscosity on the pressure fields that develop within the stomach's middle plane at *t*+ 10 s.

The pressure fields predicted by the model are illustrated in Figure 13. As expected, the pressure of the region distal to the propagation of the ACW increases significantly, and its difference with the pressure of the region behind the wave became increasingly higher as the ACW approach the pylorus sphincter. This general behavior of the pressure field is in good agreement with experimental data (Indireshkumar and others 2000; Hausken and others 2002).

Although the general behavior of the pressure field within the stomach was relatively independent of the rheological properties of the gastric contents, the pressure gradients developed were not. As illustrated in Figure 13, an increase of 1000-fold in the viscosity of the fluid lead to pressure differences within the stomach of up to 3 orders of magnitude bigger. This significant variation in the pressure field may have a critical role in promoting the gastric digestion of high viscous meals. The higher pressures may not only improve the breakdown of food particles, but also modify the motor activity of the stomach wall by increasing the distension of its wall (Marciani and others 2001c; Gregersen and others 2002).

Similar to the case of the velocity and vorticity fields, although a good qualitative agreement was found between the pressure profile predicted by this model and that predicted by Pal and others (2004), the magnitudes of the gradients predicted by the 3-D were one order of magnitude higher. Based on the previous discussion, this particular difference may have a critical effect when modeling the digestion process of the meal.

#### Limitations on the quantitative validation of the pressure field

High-resolution manometry has been lately used to locally evaluate the antroduodenal pressures associated with the ingestion of different liquid meals (Indireshkumar and others 2000; Hausken and others 2002). The experimental information gathered by these studies showed the complex pattern and variability of the pressure field within the stomach. In particular, the time history of the average pressure within the antrum and pyloric regions is characterized by a series of intermittent spikes of extremely different amplitude. The average pressure that develops within the antropyloric region varies significantly among different subjects, with values ranging between 5 and 25 mmHg and with 44% of the contractions not being detected by manometry (Indireshkumar and others 2000; Hausken and others 2002).

Although the preceding information has been used to develop a better understanding of the relative contribution of antroduodenal pressure differences to gastric emptying, the possibility of using it to validate the predicted pressure field is compromised by a series of experimental and numerical factors.

From an experimental point of view, one of the main limitations is the invasive nature of the pressure measurement technique. The disturbance that the manometric assembly may cause in the normal physiology and motility pattern of the stomach, together with the low level of pressures to be measured, may have a critical impact on the accuracy of the experimental data (Feinle and others 1999; Choe and others 2001; Pal and others 2004; Simonian and others 2004). In addition, due to the lack of a suitable reference for the absolute pressure field within the stomach, the scope of the numerical results is also limited. Since any of the prescribed boundaries included a pressure condition, to keep the pressure field from floating, the pressure value at a given reference location within the stomach model has to be subtracted from the entire pressure field obtained after each time step (Anonymous 2007). Therefore, to determine the absolute pressure field within the stomach, it is essential to define this reference location at a point where the time history of the absolute pressure is known. Due to the experimental difficulty in gathering this information from *in vivo* trials, the absolute pressure field within the stomach could not be computed.

The only possibility to quantitatively validate the pressure field in this study would be by comparing the pressure gradients that develop within the antropyloric region. However, the significant variability in these gradients made this approach meaningless. In particular, the range of antropyloric pressure gradients experimentally measured varied between 0 and 130 mmHg, more than 8 times the average pressure recorded inside the stomach (Indireshkumar and others 2000).

## Conclusions

The use of CFD provided a unique insight into the fluid dynamics of gastric contents. In agreement with the classical description of the stomach functions, the strongest fluid motions were predicted in the antropyloric region, and an important recirculation of gastric contents from the fundus toward the antrum was also identified. However, for a given motor response of the stomach, the viscosity of the gastric digesta significantly affected the local flow behavior and pressure gradients that developed within the stomach.

Contrary to the traditional idea of a rapid and complete homogenization of the meal, gastric contents associated with high viscous meals seem to be poorly mixed. By increasing the viscosity of gastric contents, the formation of the 2 main flow patterns commonly regarded as the main mechanisms driving gastric digestion (that is, the retropulsive jet-like motion and eddy structures) was significantly diminished, while a significant enhancement of the pressure field was predicted. These results, which are in good agreement with experimental data previously reported in the literature, suggest a significant role of the pressure field on the digestion process of high viscous meals.

This study also allowed the identification of a series of key factors that need to be experimentally analyzed to improve the model capabilities. In particular, it is essential to acquire a better understanding of the physicochemical properties of gastric digesta and the associated motor response of the stomach, for different types of diet.

Future work needs to be done to perform a quantitative validation of the developed model. In particular, this study illustrates the possibility of using a nonintrusive flow measurement technique, for example, particle imaging velocimetry, to trace the flow field that develops within a closed system aimed at simulating the peristaltic movement of the stomach wall. To quantitatively validate the pressure field, it is essential to identify a specific location within the stomach to experimentally determine the pressure history during digestion, and use it as a reference for numerical computations. This location should be such that it minimizes the dependency of the pressure measurements on the shape, size, and motor response of the stomach during digestion.

From a broader perspective, this study illustrates the capability of CFD to provide a unique insight into the fluid dynamics of the gastric contents, pointing out its potential to develop a fundamental understanding and modeling of the mechanisms involved in the digestion process.

## Nomenclature

*k*: consistency index, Pa.s.
*n*: flow behavior index.
*P*: static pressure, N.m^−2^.
*t*: time (s).
*u_i_*: velocity component in *x_i_* direction, m/s.
*w*: vorticity magnitude , 1/s.
*x_i_*: Cartesian tensor notation of the spatial coordinates.
*x*, *y*, *z*: spatial coordinates, m.

**Greek symbols**
shear rate, s^−1^. Related to the 2nd invariant of the rate of strain tensor (*S_ij_*) as follows:
μ_*a*_: dynamic viscosity, Pa.s.
ρ: density, kg.m^−3^.
